# Climate and Pest-Driven Geographic Shifts in Global Coffee Production: Implications for Forest Cover, Biodiversity and Carbon Storage

**DOI:** 10.1371/journal.pone.0133071

**Published:** 2015-07-15

**Authors:** Ainhoa Magrach, Jaboury Ghazoul

**Affiliations:** Institute of Terrestrial Ecosystems, ETH Zürich, CHN G 73.1 Universitätstrasse 16, 8092 Zürich, Switzerland; Institute of Agronomy, University of Lisbon, PORTUGAL

## Abstract

Coffee is highly sensitive to temperature and rainfall, making its cultivation vulnerable to geographic shifts in response to a changing climate. This could lead to the establishment of coffee plantations in new areas and potential conflicts with other land covers including natural forest, with consequent implications for biodiversity and ecosystem services. We project areas suitable for future coffee cultivation based on several climate scenarios and expected responses of the coffee berry borer, a principle pest of coffee crops. We show that the global climatically-suitable area will suffer marked shifts from some current major centres of cultivation. Most areas will be suited to Robusta coffee, demand for which could be met without incurring forest encroachment. The cultivation of Arabica, which represents 70% of consumed coffee, can also be accommodated in the future, but only by incurring some natural forest loss. This has corresponding implications for carbon storage, and is likely to affect areas currently designated as priority areas for biodiversity. Where Arabica coffee does encroach on natural forests, we project average local losses of 35% of threatened vertebrate species. The interaction of climate and coffee berry borer greatly influences projected outcomes.

## Introduction

Climate change projections suggest that many currently cultivated areas will become less suitable for agriculture, at least for currently planted crops [1, 2]. There is particular concern for coffee [3–6], the production of which is highly sensitive to local climate [7–10]. This has broad implications in that coffee is one of the world’s most important crop commodities, worth around $15 billion a year [11], and is grown by more than 25 million farmers in 60 countries [12]. While IPCC projections encompass a global temperature rise of 2–4°C by 2081 [13] and, more controversially, increased frequency and intensity of precipitation extremes [14], regional warming and increasingly erratic rainfall have already increased the frequency of poor harvests [15], and this has affected coffee prices regionally and even globally. Moreover, the area suitable for wild *Coffea arabica* L. (Arabica coffee), already restricted to small areas of Ethiopia, South Sudan and Kenya, is expected to diminish and even disappear [16], and with it much of the species’ genetic variability.

Farmers rely almost entirely on two coffee species, *Coffea arabica* L. (Arabica coffee) and *Coffea canephora* Pierre ex Froehner, syn. *Coffea robusta* (Robusta coffee). Arabica coffee grows well in a relatively narrow range of climatic conditions, including temperatures of 15 to 24°C, though best production is achieved at 18 to 22°C. Robusta coffee is hardier at higher temperatures, and is productive up to 30°C, with optimum production between 22 and 28°C [17]. Outside their optimum temperature ranges, the bean quality of both species declines, as does yield. Changing climate might also increase exposure and vulnerability of coffee to pests and diseases. The coffee berry borer, for example, is expected to spread into higher latitudes and altitudes under a warmer climate [18]. In view of changing climates and pest pressures, future suitable areas for coffee production might not coincide with currently planted areas.

Like many other commodity crops, the expansion of coffee has historically led to direct and indirect deforestation with important social and environmental impacts [19–21]. This historical precedent suggests that continued expansion of coffee to meet increasing global demand (following a ~0.1% annual increase in the surface devoted to the crop between 1975 and 2012, [22]), will likely come at the expense of some forest loss. The extent to which this is realised will, however, differ depending on where this expansion takes place (as suggested for oil palm, [23]). Moreover, future forest losses due to coffee production might exceed that expected by expansion alone if climate change forces shifts in coffee cultivation from presently cultivated areas to new areas. A spatially-explicit analysis of suitable coffee growing areas under future scenarios of climate change is needed to map possible shifts in the distribution of coffee cultivation. By identifying coffee plantation areas likely to be most vulnerable to climatic changes, such analyses might allow coffee producers to anticipate and adapt to future climatic threats to coffee production. Moreover, we can also use scenario analyses to identify areas most suited to future coffee production, and evaluate potential impacts on forests and biodiversity of the expansion of coffee in these regions. To this end we use ecological niche modelling to map areas suitable for coffee production under climate scenarios for 2050 to ascertain whether there is sufficient land available to meet future demands for coffee, and to evaluate trade-offs with alternative land uses, including deforestation, and associated costs to carbon and biodiversity.

## Results

**Fig 1:**
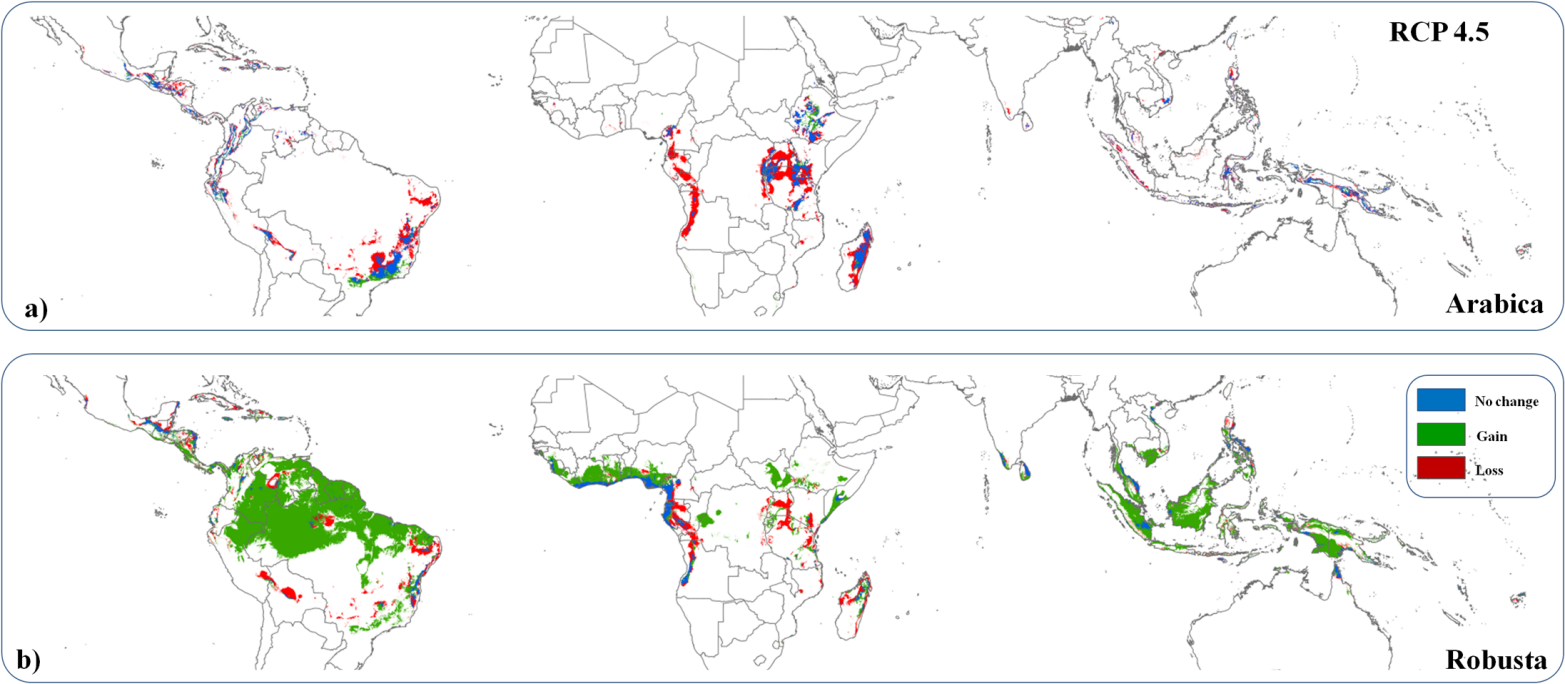
Projected suitability for a) Arabica and b) Robusta coffee cultivation in 2050 under one of the possible scenarios based on greenhouse gas emissions (RCP4.5) compared with the present area suitability and showing loss, gain and no change in suitability for each grid cell.

Our models (S1 and S2 Figs for full and pruned models, S2 Table) suggest that climate change will have disparate effects for the two species of coffee considered. At least 83% (±4%, mean ± SD for four concentration pathways considered) of the total future area suitable for coffee matches conditions required for Robusta cultivation, but only 17% (± 6%) of this area meets the requirements for Arabica (Table 1). The Arabica variety could lose 56% (± 7%) of the areas currently suitable for its cultivation by 2050 (particularly in Brazil, East Africa, and Madagascar), with only a small gain of new suitable areas (9% (± 1%), mostly within Sudan and southern Brazil, Fig 1A), totalling 19.4 (± 2.6) million hectares suitable for the crop. The Robusta variety could lose 55% (± 6%) of currently suitable areas (mostly within western Africa and Brazil), but the future suitable area is expected to more than double, particularly by the extension of climatically suitable conditions in the Amazon Basin and South East Asia (Fig 1B) which together will total 97.4 (± 16) million hectares (Table 1).

**Table 1:** Area suitability for coffee cultivation under the four concentration pathways of projected climate change in 2050 showing total suitability across all habitat types, suitability within areas not currently covered by forest, those not covered by other crops and in particular by cocoa and separating between the surface that will be suitable for the two main species of coffee. Bold numbers indicate surfaces that are not enough to meet future demand, with total future demand for coffee being 10,507,974 and that for Arabica being 7,355,581. All areas are in hectares.

Concentration pathway	Coffee species	Total area suitable	Suitable area outside forest	Suitable area outside forest and other crops
**RCP26**	Arabica	21,288,000	10,652,800	9,106,176
	Robusta	76,247,500	27,864,000	21,105,850
**RCP45**	Arabica	18,744,200	9,876,630	8,496,244
	Robusta	112,722,000	38,658,700	29,496,392
**RCP60**	Arabica	21,628,100	11,119,300	9,632,061
	Robusta	93,171,400	35,325,700	27,198,722
**RCP85**	Arabica	16,011,900	7,808,080	**6,731,676**
	Robusta	107,420,900	34,399,300	25,622,863
***Average***	*Arabica*	*19*,*418*,*050*	*9*,*864*,*203*	*8*,*491*,*539*
	*Robusta*	*97*,*390*,*450*	*34*,*061*,*925*	*25*,*855*,*957*
***SD***	*Arabica*	*2*,*610*,*043*	*1*,*463*,*438*	*1*,*261*,*706*
	*Robusta*	*16*,*334*,*943*	*4*,*518*,*686*	*3*,*543*,*710*

**Fig 2:**
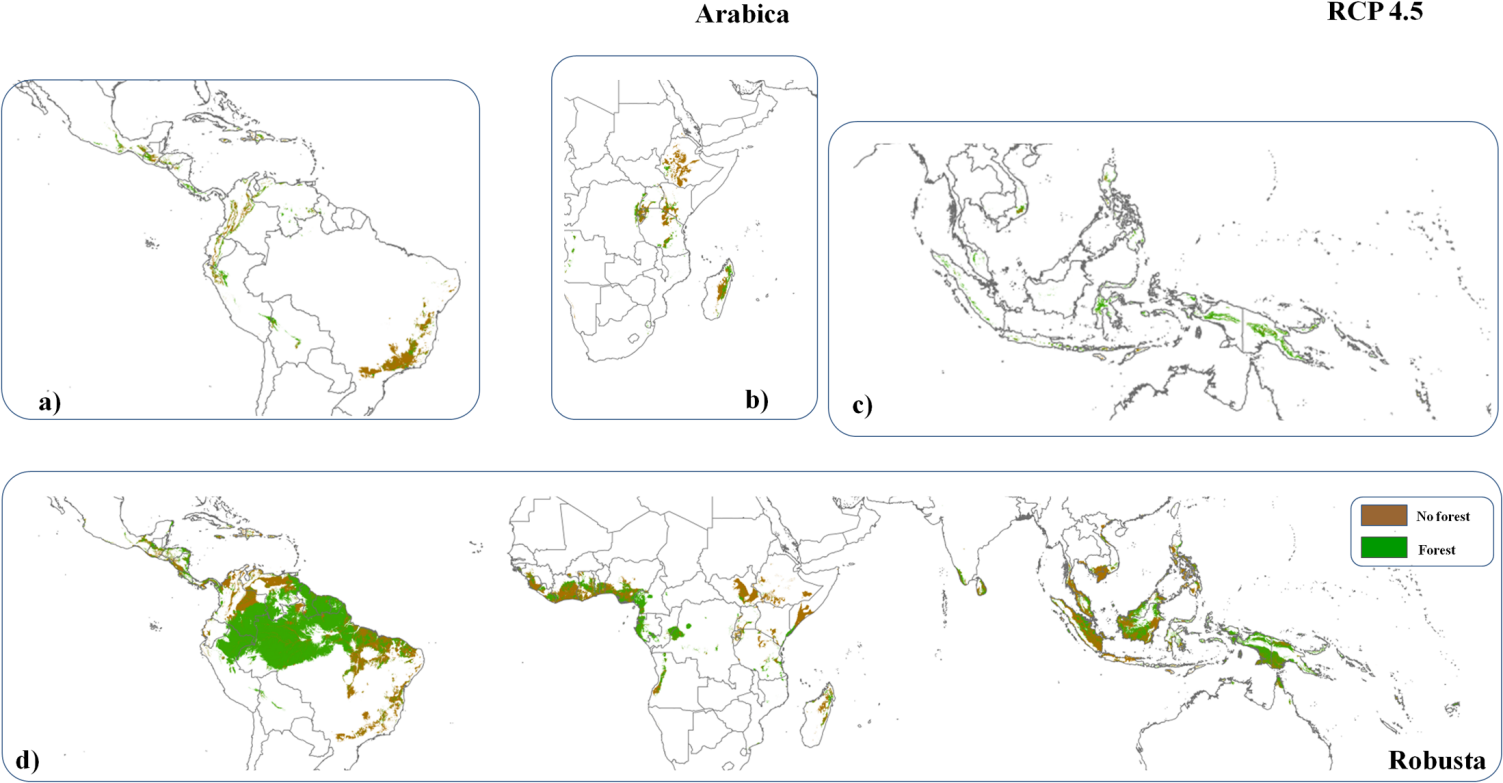
Global distribution of optimal areas for a)-c) Arabica and d) Robusta coffee plantation in 2050 predicted by the model HADGEM2-AO and current forested surfaces, showing areas where coffee suitability might conflict with forest presence.

These figures represent enough suitable area to meet future demands. Yet, 49% (±2%, 9.5 ± 1.2 million hectares) of the future area suitable for Arabica cultivation, and 65% (±12%, 63.33 ± 12 million hectares) of that for Robusta, are under forest cover (e.g., within the Amazon Basin, Indonesia, Papua New Guinea, Cameroon, Gabon, and Congo, Fig 2A–2D). This compares to 37% and 42% of current suitable area for Arabica and Robusta respectively which is under forest. Moreover, 14% (±0.1%) of future area suitable for Arabica (1.4 ± 0.21 million hectares) and 24% (± 2%) of area suitable for Robusta (8.2 ± 1.05 million hectares) are currently under the cultivation of other crops.

**Fig 3:**
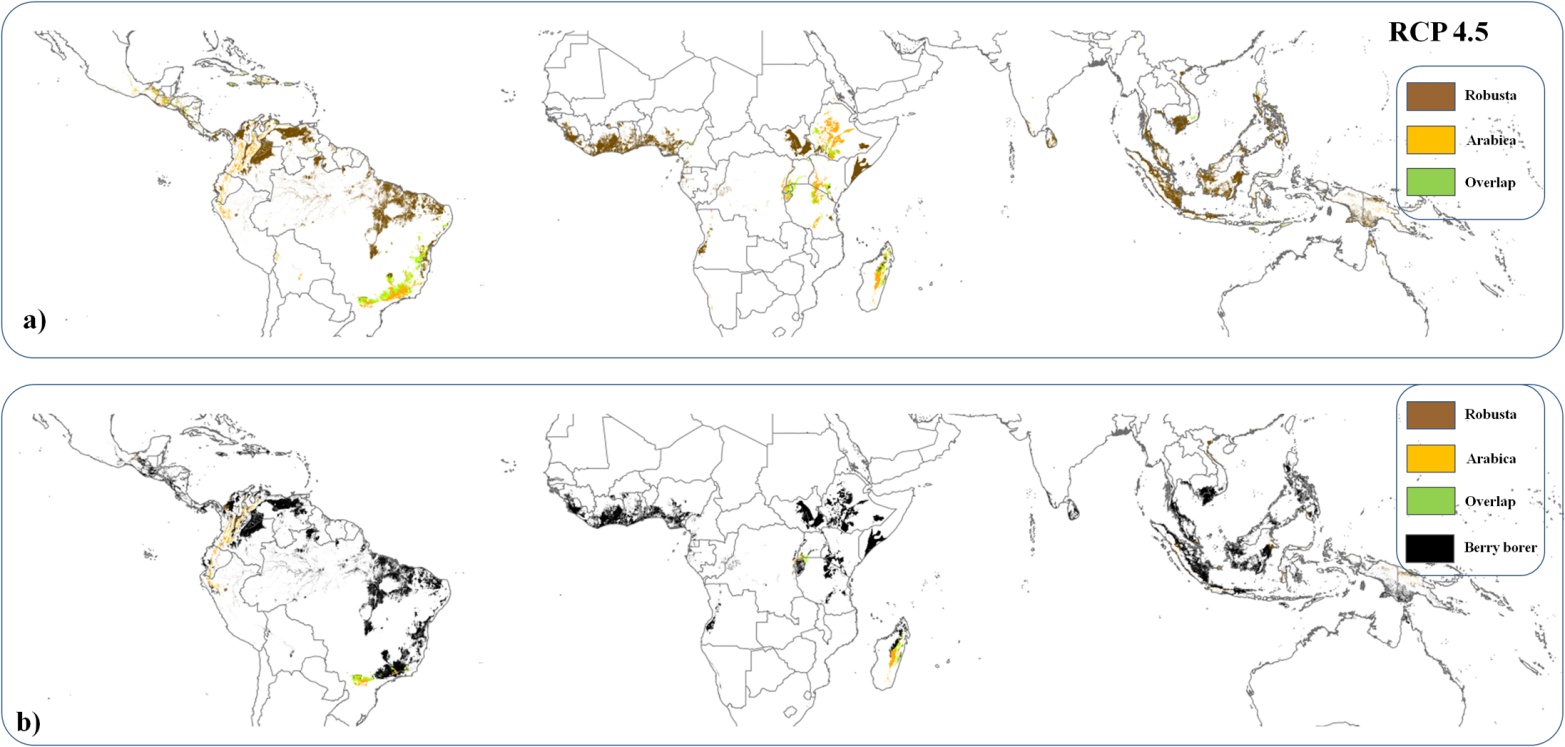
a) Global distribution of optimal areas for coffee cultivation in 2050 predicted by the model HADGEM2-AO separating between those suitable for Arabica and Robusta cultivation after accounting for areas currently covered by forest and other crops and b) future distribution of their main pest the coffee berry borer under the RCP4.5 scenario.

Confining the distribution of optimal coffee cultivation to areas that are not currently covered by either forest or other crops (particularly cocoa for which global demand is expected to increase greatly) reduces future suitable area availability for Arabica coffee to 8.49 (± 1.26) million hectares (Table 1, Fig 3A). This would be sufficient to meet future demands under three scenarios. Nonetheless, under one of the modelled concentration pathways (RCP8.5), only 6.73 million hectares will be suitable for cultivation outside of forested areas and those covered by other crops. These areas are not sufficient to meet future coffee demands (Table 1), requiring the transformation of 0.62 million hectares of forested areas.

In the case of Robusta, the area suitable for coffee excluding forest and other crops (25.8 ± 3.5 million hectares, Table 1) would be more than enough to meet future expected demand (3.2 million hectares at 30% of global coffee production, Fig 3A). Most of this area is currently classified as shrub and herbaceous cover. Nonetheless, these figures mask important regional shifts in coffee cultivation. Notably, suitable area for coffee is projected to decrease substantially in Brazil, which currently accounts for 35% of global production, and might lose up to 84% of current cultivated areas.

### Impacts of pest pressure

Area requirements for coffee cultivation might be increased subject to the responses of pests to future climates. The coffee berry borer (S3 and S4 Figs for full and pruned models, S2 Table) is projected to expand its distribution under the four possible climate trajectories, potentially affecting 77.8 ± 1.7% of Arabica and up to 93.02 ± 1.3% of Robusta plantations (Table 2, Fig 3B), as compared to ~57% and 50% of coffee suitable areas for Arabica and Robusta respectively that are currently exposed. Under an 8% attack rate scenario, only one of the four climate models predicted a net forest loss for Arabica, of 1.2 million ha. Under a 24% attack rate scenario the four climate models predicted a net forest loss for Arabica, of 0.43, 0.9 million, 0.035 million and 2.24 million ha (Table 2). It should be noted that these are best-case scenarios in that they assume the complete conversion of suitable areas of non-forest and non-crop land before any encroachment into forest. The area needed in 2050 for Robusta cultivation can be met under every scenario without incurring encroachment into forest or crop lands, even given increased exposure to the berry borer (Table 2).

**Table 2:** Percent surface attacked by the coffee berry borer for each of the main coffee varieties and surface left after attack under an 8% or 24% attack rate by the berry borer and potential forest surface that might need to be changed to coffee cultivation in order to meet future demand. For areas where forests might have to be changed to coffee we show values of average numbers of threatened species of vertebrates and conservation templates found within each grid cells, values show average and standard deviation for 100 simulations of coffee expansion.

**8% attack rate**							
	**Coffee species**	**% Surface attacked**	**Surface left after attack**	**Forest loss**	**Threatened species (mean ± SD)**	**Conservation templates (mean ± SD)**	**Carbon content (t/ha)(mean ± SD)**
**RCP26**	Arabica	76.30	8,377,682	0			
**RCP26**	Robusta	91.20	19,417,382	0			
**RCP45**	Arabica	76.61	7,816,544	0			
**RCP45**	Robusta	93.47	27,136,681	0			
**RCP60**	Arabica	79.80	8,861,496	0			
**RCP60**	Robusta	94.20	25,022,824	0			
**RCP85**	Arabica	78.80	6,193,142	**1,162,439**	**19 ± 9.51**	**5 ± 1.2**	**98.4 ± 56.8**
**RCP85**	Robusta	93.20	23,573,034	0			
**24% attack rate**							
	**Coffee species**	**% Surface attacked**	**Surface left after attack**	**Forest loss**	**Threatened species (mean ± SD)**	**Conservation templates (mean ± SD)**	**Carbon content (t/ha)(mean ± SD)**
**RCP26**	Arabica	76.30	**6,920,693**	**434,888**	**19 ± 9.65**	**5 ± 1.21**	**93.90 ± 54.77**
**RCP26**	Robusta	91.20	16,040,446	0			
**RCP45**	Arabica	76.61	**6,457,145**	**898,436**	**19 ± 9.6**	**4.00 ± 0.7**	**98.97 ± 54.74**
**RCP45**	Robusta	93.47	22,417,258	0			
**RCP60**	Arabica	79.80	**7,320,367**	**35,214**	**18 ± 9.77**	**5.00 ± 1.24**	**102.28 ± 58.88**
**RCP60**	Robusta	94.20	20,671,028	0			
**RCP85**	Arabica	78.80	**5,116,074**	**2,239,507**	**19 ± 9.53**	**5 ± 1.2**	**98.24 ± 56.71**
**RCP85**	Robusta	93.20	19,473,376	0			

### Impacts on vertebrates, priority conservation areas, and carbon

**Fig 4:**
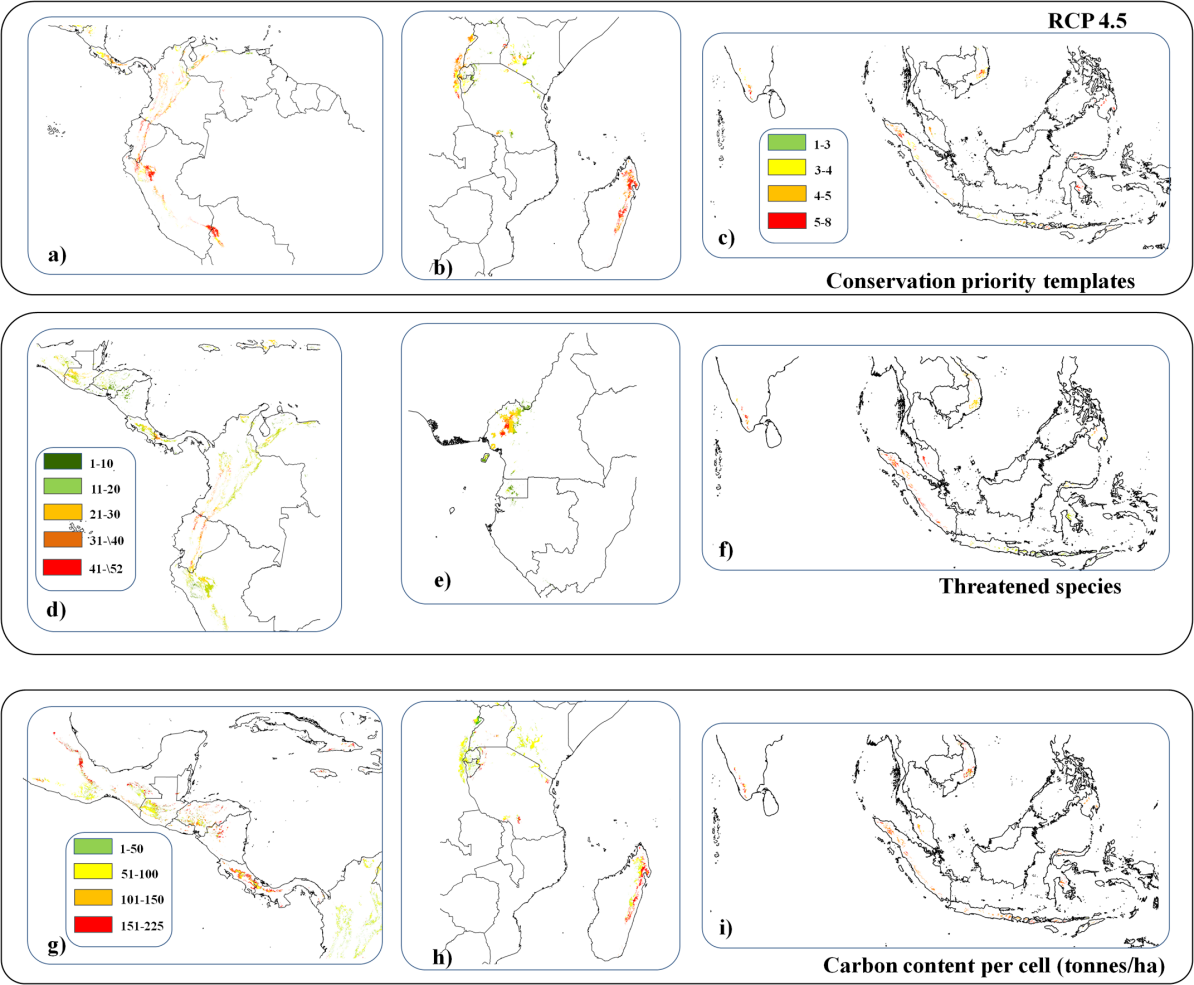
Subset of areas that will be suitable for Arabica cultivation in the future, that are currently under forest cover and within the vicinity of areas covered by coffee plantations at present showing a) the number of conservation priority templates that coincide with them, b) the number of threatened vertebrate species they host, and c) the amount of carbon stored per ha in the forests.

Encroachment of coffee into forested areas within the five scenarios in which the area suitable outside of forested areas will not be sufficient to meet future demands for Arabica (RCP 8.5 under an 8% berry borer attack rate and RCP2.6, RCP4.5, RCP6.0 and RCP8.5 under a 24% attack rate, Table 2) would affect a grid cell average of almost five priority conservation areas (Table 2, Fig 4A), and an average of 19 threatened vertebrate species (Table 2, Fig 4B). The greatest impacts on conservation priority areas would occur in Peru, Bolivia, Madagascar, Indonesia and India (Fig 4A). Coffee expansion would impinge most severely on vertebrate diversity in Costa Rica, Ecuador, Peru, Cameroon, Indonesia, Malaysia and India (Fig 4B) due to the large number of threatened (and endemic) species.

The conversion of forest to coffee plantations is expected to cause a loss of 5.90 to 15.66 million tonnes of carbon if forested grid cells were converted to shade or sun coffee plantations respectively (Table 2, Fig 4C, under scenario RCP4.5). Major carbon losses would occur in Panama, Costa Rica, Nicaragua, Mexico and, above all, Madagascar, Indonesia and India (Fig 4C).

## Discussion

Our projections indicate that climate change will be detrimental for Arabica cultivation, though the area suitable for Robusta will increase greatly by 2050. Both Arabica and Robusta will be subject to important geographic shifts in their distribution. Many projected coffee-optimal areas will coincide with existing forest and agricultural land uses, including cocoa, demand for which is expected to increase more quickly than for coffee. The future projected distribution and expansion of Robusta coffee-growing areas can potentially be accommodated without incurring deforestation or crop substitution, as climatic conditions across most of the future suitable coffee-growing area favour the hardier Robusta variety. In contrast, only 17% of this area is optimal for Arabica. This raises some concern as Arabica is the preferred variety, and currently accounts for 70% of global production [24]. Increasing demand for Arabica could, therefore, lead to forest clearance as conditions for its cultivation will be spatially more restricted, thus constraining land allocation options. The extent of forest loss to Arabica cultivation will be mediated by the coffee berry borer, which can cause up to 24% crop loss. Under such circumstances we project conversion of up to 2.2 million hectares of forest to coffee plantations to meet Arabica expected demands. This has corresponding implications for carbon storage, and will affect areas currently designated as biodiversity priority areas, and might mean projected local losses of 35% of threatened vertebrate species on average.

While our results suggest that the area suitable for both Arabica and Robusta cultivation will be sufficient under some climate change scenarios to match future expected demand, there will be regional shifts in the global suitability for coffee (as suggested by [6]). In particular, the suitability for future coffee cultivation of Brazil, currently the world’s largest producer is projected to decline. This result is driven by increases in rainfall and temperature, coupled with increased seasonality, drier dry seasons, and wetter and warmer wet seasons [9, 10]. Such changes might be mitigated through irrigation and increasing tree shade over the currently open sun plantations that constitute the common system of coffee cultivation in these areas. Shade tree management alleviates temperature and drought stresses, and can also reduce coffee berry borer impact [25]. Continued climate change in this region coupled with extreme events might ultimately favour a shift to other less sensitive crops, with oil palm being one possible candidate [22].

Many areas of projected increased coffee cultivation suitability will be located in currently forested locations, particularly in the Amazon Basin, Indonesia, Papua New Guinea, Cameroon, Gabon, and Congo. This creates at least the potential for forest conversion, typical of recent trends in agricultural expansion in the past decades [19–21]. Encroachment of coffee into forested areas under the above-mentioned scenarios will have implications for threatened vertebrate species and conservation areas. By assigning values to each grid cell for the number of threatened species, and number of overlapping priority areas for conservation, we project that up to 2.2 million hectares of forested area might be needed for Arabica development (RCP8.5 concentration pathway and 24% berry borer attack) and this would affect large numbers of threatened vertebrate species and impact upon important priority conservation areas with associated losses of significant values of carbon stocks.

Whether this happens depends on several factors, including whether farmers in these regions switch from current crops to coffee and, if they do, whether there is leakage through the displacement of former crops into forested areas [19]. Moreover, farmers’ decisions to cultivate coffee on existing cultivated lands will also be shaped by commodity market prices of coffee relative to other locally suitable crops such as cocoa or oil palm. Even if coffee cultivation is accommodated on non-forested and non-crop lands, its development might nevertheless incur conflicts with other land cover types which may in themselves have biodiversity or other resource or ecosystem service values, which should be evaluated at local scales (e.g., if it moves into higher altitudes as suggested by [6]).

The projected impacts to forest cover, biodiversity and carbon will additionally be shaped by land use and management decisions made by coffee cultivators. Shade tree cover might mitigate adverse climatic effects by alleviating temperature and drought stresses [26], and can also reduce coffee berry borer impact [25]. Moreover, coffee under native shade trees can support substantial biodiversity within modified landscapes [27]. Although coffee grown under shade is generally less productive [28], farmers are more likely to avoid vulnerabilities associated with climatic extremes and biotic pressures through shade tree management.

Our study is subject to a number of caveats. We have not considered how diseases such as leaf rust (significant in Central America, [29]) or other agricultural pests and diseases (e.g., coffee berry disease or coffee leaf wilt) will respond to climate change, nor how coffee productivity might be affected by a potential CO_2_ fertilisation effect. In addition, the very short blooming period for coffee flowers (~48 hours,[30]) might be greatly affected by changing precipitation patterns, with important direct effects on flowers but also affecting their interaction with their main pollinators [31]. Further, the different coffee-growing methods used across the world might have different buffering effects against climate change and we have not accounted for this within our modelling. These remain major uncertainties for model development. Further, although our models suggest that the availability of suitable areas for Robusta cultivation might mitigate the area losses for Arabica production, this will ultimately depend on the choice of consumers.

A recent global evaluation of the effect of climate change on coffee distribution [6] suggests that Robusta coffee might need to be grown in areas with little intra-seasonal variation of rainfall. Therefore, although increasing temperatures might favour this variety, the potential for increased variability (i.e., more extreme events) in weather conditions might have negative consequences for Robusta coffee that our models have not anticipated. This implies that our models might overestimate the area suitable for Robusta.

We have also not taken account of the allocation of land to other crops. Whether coffee expansion occurs at all in non-forested areas depends on the accessibility of land for cultivation, and opportunity costs of coffee relative to other land uses. Oil palm and, to a lesser extent, cocoa, are particularly relevant in this context as these crops are undergoing rapid expansion in all main tropical regions where coffee is grown [22]. Despite these caveats, our projections suggest that the considerable extent of future suitable land area is likely to be sufficient to accommodate Robusta cultivation, despite oil palm or other crop expansion. This is not the case for Arabica, for which land use conflicts or crop trade-offs are expected.

Our results imply that moderate climate change will increase the area suitable for Robusta coffee cultivation across the globe, but decrease it for Arabica. There will also be major regional shifts, and some of the world’s largest producers (e.g., Brazil) might have to adopt shade management and irrigation to mitigate climatic stresses. At a global level, meeting future Robusta demand can, theoretically, be met without deforestation. Increasing demand for Arabica, by contrast, will very likely drive forest conversion. Forest losses will negatively impact threatened vertebrate species and priority conservation areas, and result in carbon emissions. The magnitude of such changes will be mediated by the effects of coffee berry borer under new climatic conditions. Moreover, sparing forested areas from transformation to coffee might require establishing coffee in areas that have not traditionally been associated with coffee cultivation, and will therefore depend on the willingness of local farmers to adopt an unfamiliar crop.

## Methods

### Ecological niche modelling

We randomly extracted 10,000 where coffee is currently present (including both *C*. *arabica* and *C*. *canephora*) from a geospatial dataset at 5 arc-minute resolution (~10 x 10 km grid cell) that represents the year 2000 [32]. Localities were randomly extracted but we allowed for only one locality to be used per 1 km^2^ grid cell to avoid a bias in model accuracy which could arise from the spatial autocorrelation of points located at smaller distances [33]. We then used MaxEnt (version 3.3.1, www.cs.princeton.edu/~schapire/maxent/, accessed October 2013) to relate presence data with environmental layers. We used Maxent as a modelling approach due to its use of presence-only data because although we would be able to extract absence points from the same spatial layer from which we extracted presences, absences in many cases are not real absences, meaning that the species cannot survive under the environmental conditions present there but rather an artificial consequence of farmer preferences. We used 80% of our locality data as a training dataset, with the remaining 20% of presence localities used as testing data to validate the models and we included 10,000 background points (or pseudo-absences) to fit models. The regularization setting was set to 2 for data smoothing while we used the default settings for the rest of parameters. We also constrained predictions outside of the limits of current climate conditions by disabling the extrapolate option in Maxent. For each model we ran 10 cross-validations in order to provide more robust estimates [34].

Environmental layers included the 19 ‘‘BIOCLIM” layers, which represent annual trends of various aspects of temperature, precipitation, and seasonality with a spatial resolution of 30 arc seconds (~1 km by 1 km [35]). In addition, we included altitude extracted from a global digital elevation model with the same spatial resolution (1 km x 1 km, [36]), a set of soil characteristics extracted from the FAO digital soil map [37], in particular: CaCO_3_, C/N ratio, clay content, nitrogen, pH, sand and silt at a 1km by 1 km resolution. Finally due to the predicted intensification of the ENSO phenomenon we included a proxy for this as the relative average annual difference in Normalized Difference Vegetation Index (NDVI) between an ENSO and a non-ENSO year (as in [38]) at a 10 km by 10 km resolution which we resampled in ArcGIS 10.0 to match the resolution of other layers as this information is currently not available in a finer resolution (see S1 Table for complete list of variables). Model accuracy was evaluated using the area under the Receiver Operating Characteristic (AUC) curve for both the training and the test data. This curve shows the relationship between sensitivity (or the proportion of presences that are correctly predicted by the model) and specificity (which represents 1 minus the percentage of absences correctly predicted). Model accuracy is considered insufficient for values ranging from 0.50 to 0.60, poor when between 0.60 and 0.70, average for 0.80–0.90 and excellent over 0.90 [39]. We first fitted a full model which included all the variables (S1 Table). This full model might be oversized, overfitted and redundant [33] and we therefore created a pruned model by retaining only the variables that contribute most to model accuracy. This was calculated using a jack-knife test within Maxent that systematically excludes the variables one at a time and calculates how much they each contribute to model precision [33]. We then used the variables that most contribute to model accuracy to fit the pruned model. In addition, we ran the same pruned model 99 times using 99 sets of 1,000 random locations (extracted from a global crop layer in which coffee was eliminated [40]) and calculated average AUC and 95% confidence interval for them and compared it to our models in order to test whether our models differed from that expected by chance [38].

### Future climate change modelling

Future models were generated using the same set of variables used for the baseline present modelling: 19 BIOCLIM layers, 7 soil variables, altitude and ENSO measure. Climatic layers were extracted from published resources which had been projected and downscaled (through interpolation to high resolution, 1 km by 1 km grids and assuming that change in climate is relatively stable across space) from different global climate models under four representative concentration pathways of the IPCC fifth Assessment Report (AR5), describing four possible greenhouse gas concentration trajectories which would lead to a range of radiative forcing values in 2100 relative to pre-industrial values: RCP2.6, RCP4.5, RCP6.0, RCP8.5, +2.6, +4.5, +6.0 and +8.5 Watts/m^2^ respectively [41]. We used HADGEM2-AO developed at the Hadley Centre [42] as global circulation model downscaled to 30 arc seconds [43]. We used this model for the time interval 2050 representing the average for 2041–2060. For each emission scenario we projected suitability for coffee plantations using MaxEnt. We calculated an optimal suitability threshold at a cut off value of 68% (roughly equivalent to one standard deviation) of the 1,000 localities considered based on their values within the niche models.

### Potential conflict between coffee and other land uses

We distinguished among areas where conditions are more suitable for Robusta or Arabica cultivation based on the average temperature of the area (BIO1 variable of the “BIOCLIM” layers), assuming Arabica supports temperatures of 15 to 24°C (with best performances at 18 to 22°C) whereas Robusta tolerates higher temperatures from 20 to 30°C (with optimum values ranging from 22 to 28°C [17]). We then evaluated the potential conflict that could exist between the expansion of coffee plantations of both species and other land uses such as forests or other crops, given the likely future increases in demand for coffee across the globe. Our projection of coffee expansion was based on a global increase of ~92% in coffee production from 1975 to 2012 (increasing from 4.6 million tonnes in 1975 to 8.8 million in 2012, [11, 22]), although with a smaller increase in area occupied by coffee cultivation which increased by 11% during this period (from 9 million hectares in 1975 to 10.04 million in 2012, an increase in surface of 0.1% per year on average) due to a strong increase of ~72% in yield, with an average 1.7% increase in efficiency for this period [22]. Therefore, assuming that coffee cultivation will increase its surface at the same 0.1% average annual rate until 2050, we expect that areas needed for coffee cultivation will increase from 10,039,846 hectares in 2012 to 10,507,974 hectares by 2050. Although we are aware that there have been decreases in the area devoted to coffee production during the same period due to important fluctuations in the coffee market and to national legislations, we believe that this kind of analysis is out of the scope of our study on the ecological implications of the effect of climate change on a particular crop at the global level. We also assumed that Arabica would represent 70% of coffee production following present consumer preferences and therefore area need for this species would be 7,355,581 hectares in 2050.

Forested areas were extracted by merging together all the natural tree cover categories from the GLC2000 global land cover map at 1 km by 1 km spatial resolution [44]. Data on crop distribution were extracted from a previous publication [40] and resampled to a 1km by 1km resolution in ArcGIS 10.0 (as they were previously available at a 10 km by 10 km resolution) to match the resolution of the other layers as these data are currently not available at finer resolutions. First, we evaluated the spatial congruence between projected areas suitable for Arabica and Robusta cultivation in 2050 and areas covered by forest, identifying areas of potential conflict between both land use types. Second, in order to evaluate whether the predicted expansion in areas devoted to coffee cultivation can be reached without clearing any forest or substituting other crops currently occupying a focal area, we limited projected coffee distribution to non-forested areas and to the regions of a grid cell not occupied by other crops. Third, the previous distribution of areas suitable for coffee was further constricted by not allowing coffee to be present at all in grid cells currently occupied by cocoa, given the likely expansion of this crop in the near future, which has already increased by an average of 3% per year since 2000 [22]. Fourth, we calculated the total area that will be suitable for Arabica and Robusta coffee cultivation outside of forested areas and those covered by other crops. Whenever this value was lower than the future expected demand for any of the two species, we further calculated the extra surface that would be needed which we assume here would be met through the transformation of natural forests.

### Berry borer ecological niche and future climate change modelling

Given the importance of the coffee berry borer, *Hypothenemus hampei* Ferrari, as a major pest to coffee crops across the globe we also used ecological niche modelling to project future areas where it will likely be present under the same set of climate scenarios we used for coffee (HADGEM2-AO,four concentration pathways, RCP2.6, RCP4.5, RCP6.0, RCP8.5). Modelling was based on 65 presence localities extracted from a published dataset [45]. Following the methodology used for coffee, we first fitted a full model which included all variables (S1 Table) and then a pruned model including only the variables that contributed most to model accuracy based on jack-knife tests (S1 Table). For each model we also ran 10 cross-validations and model AUC was also compared to the average of 99 models ran with random locations. Optimal suitability was calculated using a threshold at a cut off value of 68% (roughly equivalent to one standard deviation) of the 65 localities. We limited the projected distribution for the pest to areas with optimal suitability for coffee after removing areas covered by forest or other crops and separated between the effect it might have on Arabica and Robusta plantations.

The presence of the berry borer does not imply the loss of the whole crop: typically, coffee berry borer causes losses of 8 to, in extreme cases, 24% of production [46]. We therefore overlaid the projected distribution of the berry borer under each of the four scenarios considered with the future distribution of areas suitable for Arabica and Robusta coffee limited to regions outside of forested areas and those covered by other crops. In each case we calculated the surface that would be left free of the berry borer if 8% of the crop were lost to the pest and if 24% of the crop were affected. As in the previous sections we compared the remaining area to the expected future demand for each of the coffee species and calculated the extra area that would be needed to match demands, assuming again that this expansion would be done at the expense of natural forests.

### Impact of future coffee expansion on the conservation of natural habitats and threatened species

In the cases in which future Arabica expected demand cannot be met due to lack of available suitable surfaces or due to strong losses from berry borer attacks we evaluated the potential impact of the substitution of forest by coffee. We first extracted all areas that will be suitable for coffee under each of the climatic scenarios evaluated and that are currently covered by forests. Then, we limited these areas to the proximity (<1 km) of grid cells that are currently covered by coffee plantations, following the assumption that coffee developments will most probably be done in areas with a history of coffee growing. We then randomly chose grid cells within this subset to match extra areas needed under each scenario, repeating this 100 times to include potential variability between different areas. Each grid cell that might be transformed to coffee was then assigned a conservation value following two alternatives. On a first step we overlaid the ‘native’ and ‘extant’ range maps of 4,118 globally vulnerable, endangered and critically endangered vertebrates (including mammals, birds and amphibians [47–49]) to determine the number of ‘threatened’ species in each grid square that might be transformed to coffee. On a second step we determined the number of global conservation templates identifying each grid square as a priority for conservation including the following: crises ecoregions [50], biodiversity hotspots [51, 52], endemic bird areas [53], important bird areas [54], global 200 terrestrial ecoregions [55, 56], high biodiversity wilderness areas [57], frontier forests [58], and last of the wild [59] and finally on the density of above and below-ground living biomass carbon stored within the forests in each grid cell [60]. In addition, we calculated the total loss of above and below-ground carbon storage if forest were transformed to shade plantations, which might store up to 81 tonnes of carbon/ha or to sun plantations where carbon stored would be reduced to 22.9 tonnes of carbon/ha [61].

## Supporting Information

S1 Table List of climatic, soil and elevation predictors used to model the distribution of coffee and its main pest, the coffee berry borer at a global scale and selection of variables used in the pruned models. (DOCX)

S2 Table Relative performances of full and pruned models for global coffee and berry borer scales. Values show average ± standard deviation for 10 cross-validations.
(DOCX)

S1 Fig Results of full model for coffee with variable contribution from which predictors included in the pruned model were selected based on their contribution to model AUC in jackknife tests. “With” indicates the results of the model when only this predictor is run in isolation and “without” indicates results when that particular predictor is not included in the model. Values are the average for 10 cross-validations. Red line shows threshold at which predictor variables were selected for the pruned model.
(PNG)

S2 Fig Results of pruned model for coffee determined by jackknife tests. “With” indicates the results of the model when only this predictor is run in isolation and “without” indicates results when that particular predictor is not included in the model. Values are the average for 10 cross-validations.
(PNG)

S3 Fig Results of full model for the coffee berry borer with variable contribution from which predictors included in the pruned model were selected based on their contribution to model AUC in jackknife tests. “With” indicates the results of the model when only this predictor is run in isolation and “without” indicates results when that particular predictor is not included in the model. Values are the average for 10 cross-validations. Red line shows threshold at which predictor variables were selected for the pruned model.
(PNG)

S4 Fig Results of pruned model for the coffee berry borer determined by jackknife tests. “With” indicates the results of the model when only this predictor is run in isolation and “without” indicates results when that particular predictor is not included in the model. Values are the average for 10 cross-validations.
(PNG)
