# Supplementary material for: Climate and Pest-Driven Geographic Shifts in Global Coffee Production: Implications for Forest Cover, Biodiversity and Carbon Storage
Source: PLoS One. 2015 Jul 15;10(7):e0133071. doi: 10.1371/journal.pone.0133071 (PMC4503344; doi:10.1371/journal.pone.0133071)
Supplement: S1 Table — (DOCX) [file pone.0133071.s001.docx]

**S1 Table.** List of climatic, soil and elevation predictors used to model the distribution of coffee and its main pest, the coffee berry borer at a global scale and selection of variables used in the pruned models.

| **Abbreviation** | **Predictor variable** | **Used for model** | | |
| --- | --- | --- | --- | --- |
|  |  | **Full** | **Pruned coffee** | **Pruned berry borer** |
| Elevation | Height (m) above sea level | ▪ |  |  |
| BIO1 | Annual mean temperature (⁰C) | ▪ |  |  |
| BIO2 | Mean Diurnal Range (Mean of monthly (max temp - min temp)) | ▪ |  |  |
| BIO3 | Isothermality (BIO2/BIO7) (* 100) | ▪ | ▪ | ▪ |
| BIO4 | Temperature Seasonality (standard deviation *100) | ▪ | ▪ | ▪ |
| BIO5 | Max Temperature of Warmest Month | ▪ |  |  |
| BIO6 | Min Temperature of Coldest Month | ▪ | ▪ |  |
| BIO7 | Temperature Annual Range (BIO5-BIO6) | ▪ | ▪ | ▪ |
| BIO8 | Mean Temperature of Wettest Quarter | ▪ |  |  |
| BIO9 | Mean Temperature of Driest Quarter | ▪ |  |  |
| BIO10 | Mean Temperature of Warmest Quarter | ▪ |  |  |
| BIO11 | Mean Temperature of Coldest Quarter | ▪ | ▪ |  |
| BIO12 | Annual Precipitation | ▪ |  |  |
| BIO13 | Precipitation of Wettest Month | ▪ | ▪ |  |
| BIO14 | Precipitation of Driest Month | ▪ |  |  |
| BIO15 | Precipitation Seasonality (Coefficient of Variation) | ▪ |  |  |
| BIO16 | Precipitation of Wettest Quarter | ▪ | ▪ |  |
| BIO17 | Precipitation of Driest Quarter | ▪ |  |  |
| BIO18 | Precipitation of Warmest Quarter | ▪ |  |  |
| BIO19 | Precipitation of Coldest Quarter | ▪ |  |  |
| CaCO₃ | Average of CaCO₃ % topsoil | ▪ |  |  |
| C/N ratio | Average of carbon/nitrogen ratio topsoil | ▪ |  |  |
| Clay | Average of clay % topsoil | ▪ |  |  |
| N | Average of nitrogen % topsoil | ▪ |  |  |
| pH | Average of pH % topsoil | ▪ |  |  |
| Sand | Average of sand % topsoil | ▪ |  |  |
| Silt | Average of silt % topsoil | ▪ |  |  |
| ENSO | Relative average annual difference in NDVI between the months of an ENSO and a non-ENSO year | ▪ |  |  |
