# Supplementary material for: Climate and Pest-Driven Geographic Shifts in Global Coffee Production: Implications for Forest Cover, Biodiversity and Carbon Storage
Source: PLoS One. 2015 Jul 15;10(7):e0133071. doi: 10.1371/journal.pone.0133071 (PMC4503344; doi:10.1371/journal.pone.0133071)
Supplement: S2 Table — Values show average ± standard deviation for 10 cross-validations. (DOCX) [file pone.0133071.s002.docx]

**S2 Table**. Relative performances of full and pruned models for global coffee and berry borer scales. Values show average ± standard deviation for 10 cross-validations.

|  | **AUC** |
| --- | --- |
| **Coffee models** |  |
| Full model | 0.942 ± 0.0011 |
| Pruned model | 0.928 ± 0.0058 |
| Null model | 0.77 ± 0.007 |
| **Berry borer models** |  |
| Full model | 0.9495 ± 0.0048 |
| Pruned model | 0.8827 ± 0.005 |
| Null model | 0.67 ± 0.009 |
