# Supplementary figures and images for: Climate and Pest-Driven Geographic Shifts in Global Coffee Production: Implications for Forest Cover, Biodiversity and Carbon Storage
Source: PLoS One. 2015 Jul 15;10(7):e0133071. doi: 10.1371/journal.pone.0133071 (PMC4503344; doi:10.1371/journal.pone.0133071)

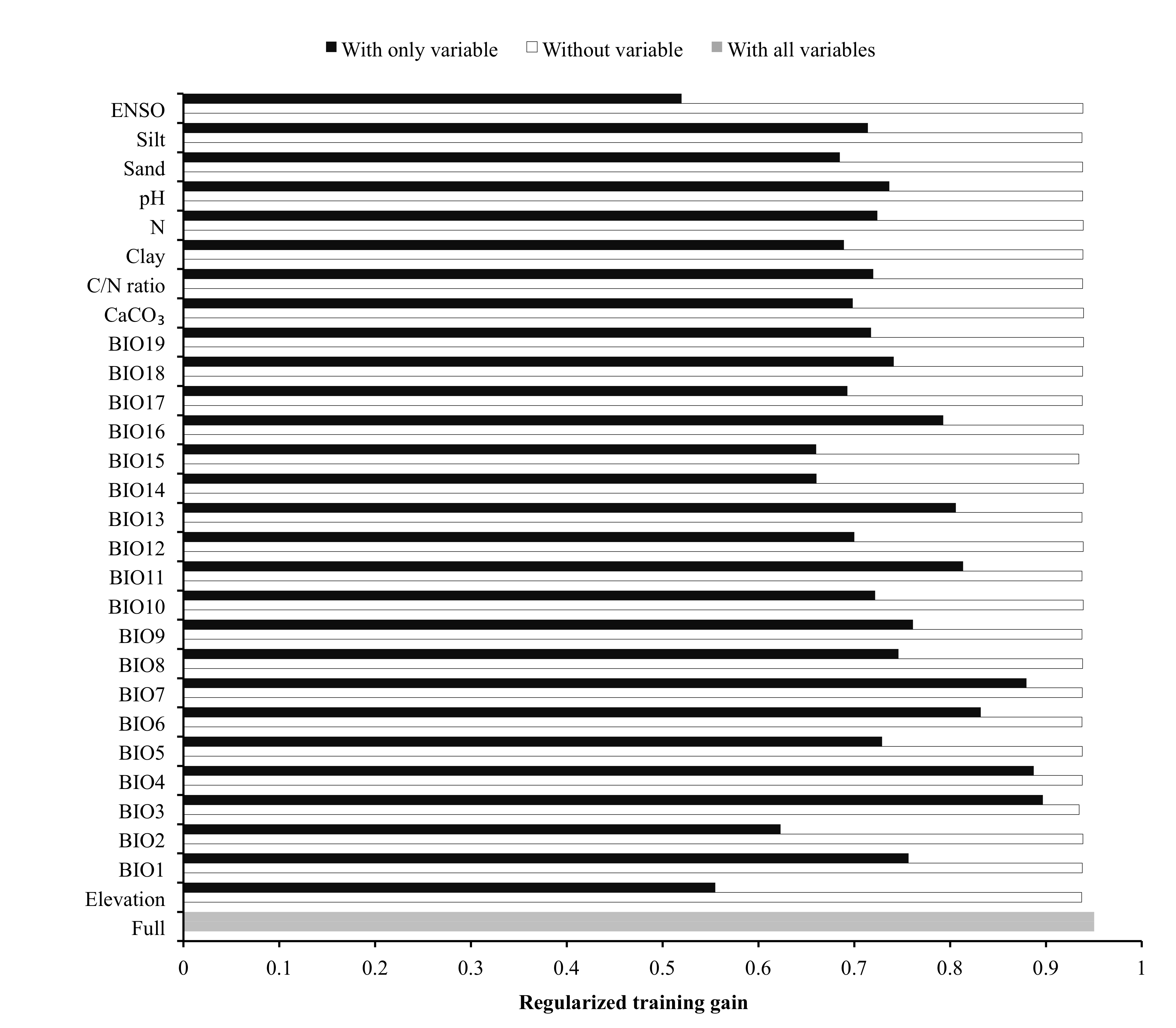

Supplement: S1 Fig — “With” indicates the results of the model when only this predictor is run in isolation and “without” indicates results when that particular predictor is not included in the model. Values are the average for 10 cross-validations. Red line shows threshold at which predictor variables were selected for the pruned model. (PNG) [file pone.0133071.s003.png]

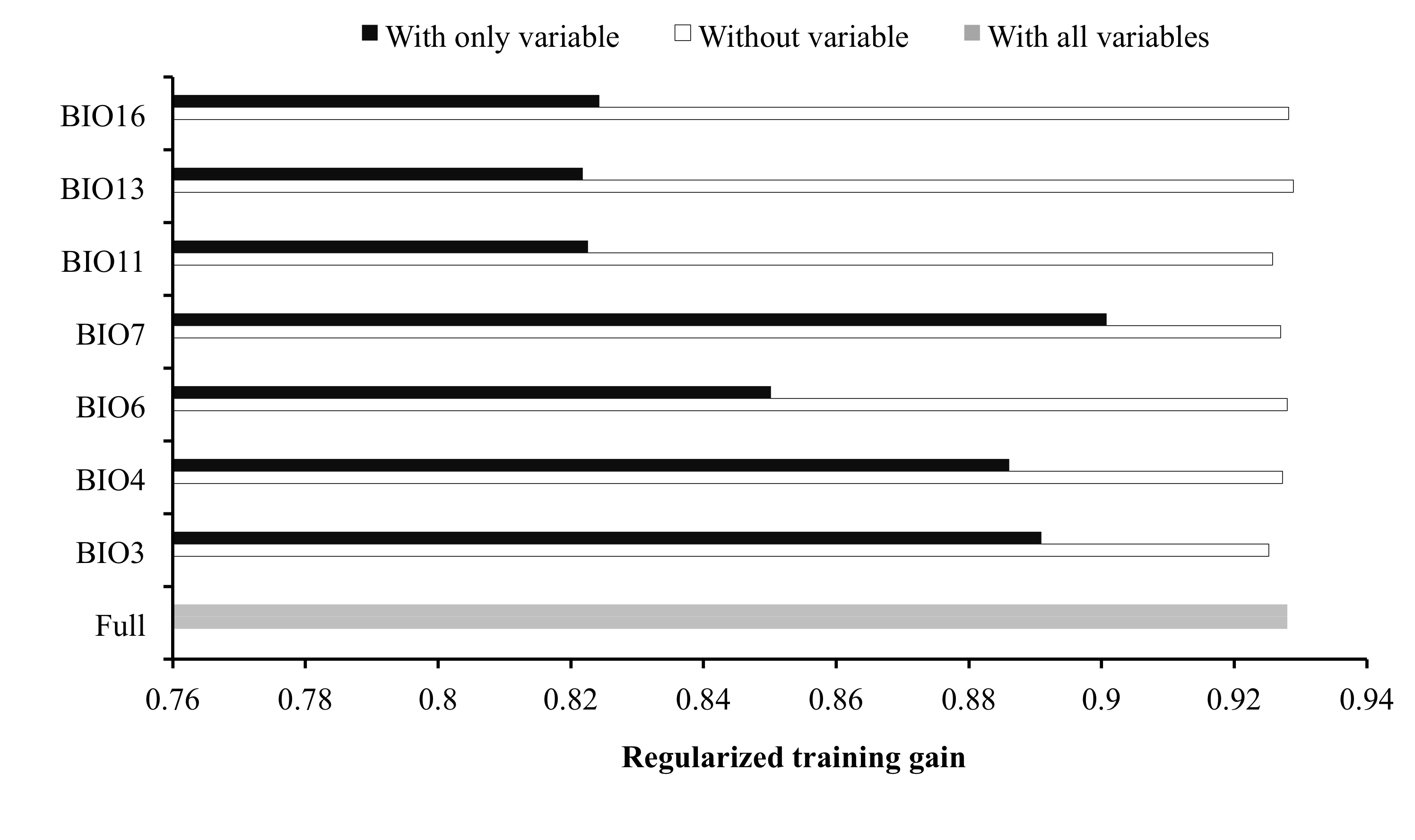

Supplement: S2 Fig — “With” indicates the results of the model when only this predictor is run in isolation and “without” indicates results when that particular predictor is not included in the model. Values are the average for 10 cross-validations. (PNG) [file pone.0133071.s004.png]

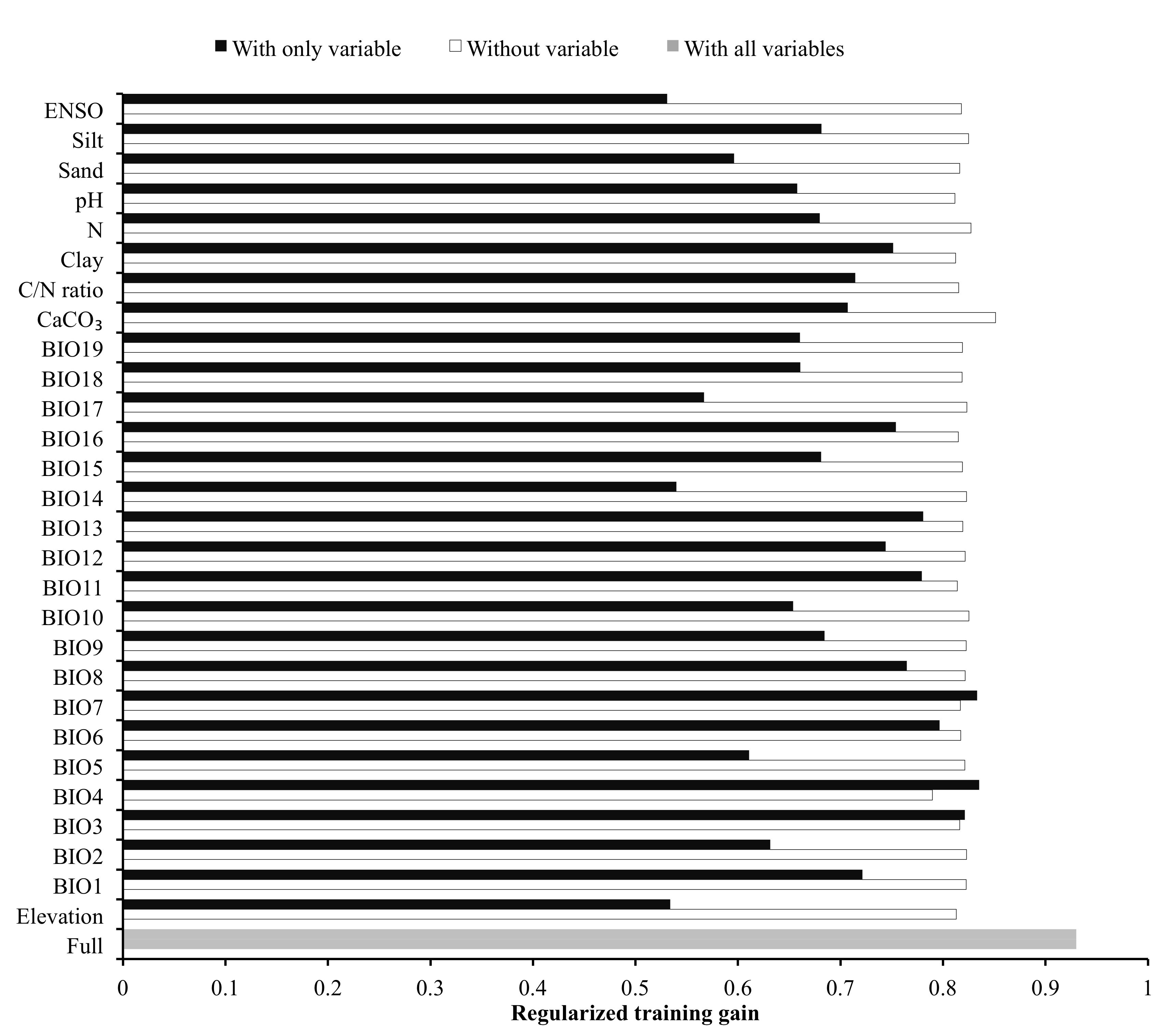

Supplement: S3 Fig — “With” indicates the results of the model when only this predictor is run in isolation and “without” indicates results when that particular predictor is not included in the model. Values are the average for 10 cross-validations. Red line shows threshold at which predictor variables were selected for the pruned model. (PNG) [file pone.0133071.s005.png]

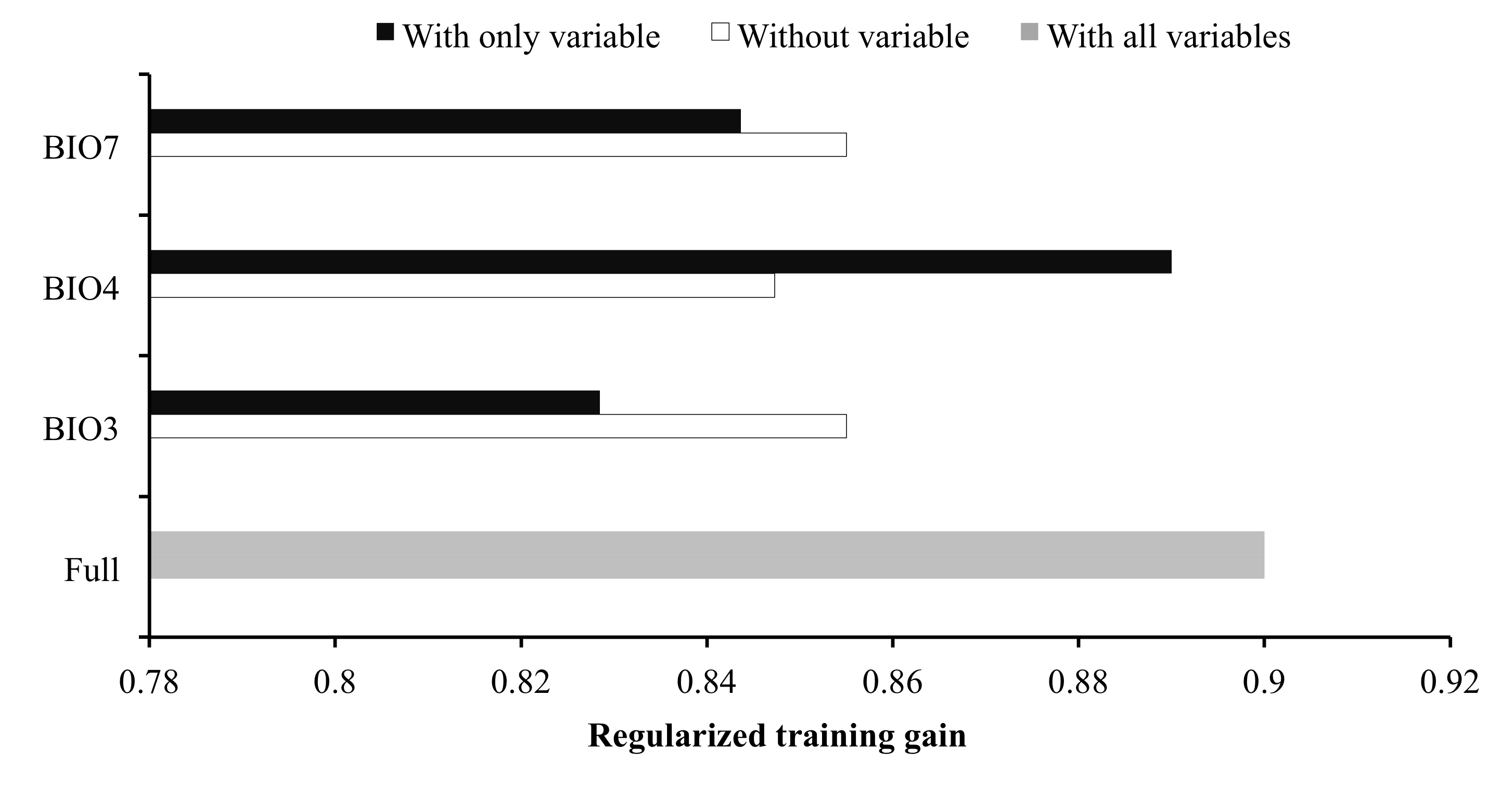

Supplement: S4 Fig — “With” indicates the results of the model when only this predictor is run in isolation and “without” indicates results when that particular predictor is not included in the model. Values are the average for 10 cross-validations. (PNG) [file pone.0133071.s006.png]
